# A Comparative Analysis of Carlevale IOL Versus Artisan IOL Implantation Using a Scleral Tunnel Incision Technique

**DOI:** 10.3390/jcm13226964

**Published:** 2024-11-19

**Authors:** Justus Obergassel, Peter Heiduschka, Florian Alten, Nicole Eter, Christoph R. Clemens

**Affiliations:** Department of Ophthalmology, University of Muenster Medical Center, 48149 Muenster, Germany; justus.obergassel@ukmuenster.de (J.O.); peter.heiduschka@ukmuenster.de (P.H.); florian.alten@ukmuenster.de (F.A.); nicole.eter@ukmuenster.de (N.E.)

**Keywords:** Carlevale intraocular lens, artisan intraocular lens, intraocular lens luxation

## Abstract

**Background:** The aim of this retrospective study was to compare the surgical and refractive outcomes using the Carlevale IOL (FIL SSF; SOLEKO) with those of the retropupillary-fixated Artisan IOL (Aphakia Model 205; OPHTEC), implanted through a 6 mm sclerocorneal tunnel incision in both groups. **Methods:** This study included 51 consecutive eyes (25 Carlevale and 26 Artisan IOLs). Due to complex preoperative conditions (e.g., dislocated polymethylmethacrylat IOL, luxated Cataracta rubra), all patients underwent lens explantation using a standardized 6 mm sclerocorneal tunnel incision and a 23 G or 25 G pars plana vitrectomy. Visual acuity (VA), spherical equivalent, refractive prediction error (PE), incision-suture time, and complication rates were recorded preoperatively and during the follow-up period. **Results:** The average follow-up period was 40.9 ± 5.7 days. VA improved by 0.28 ± 0.39 logMAR (*p* < 0.0001) in the Carlevale group and by 0.36 ± 0.47 logMAR (*p* < 0.0001) in the Artisan group. The improvement was comparable between both groups (*p* = 0.921). The deviation of the PE was −0.67 ± 0.56 in the Carlevale group and 0.34 ± 0.71 in the Artisan group (*p* < 0.0001). The mean incision-suture time was 42.5 ± 5.8 min in the Carlevale group and 28.2 ± 6.4 min in the Artisan group. Anterior chamber and vitreous hemorrhages were the most common complications, occurring in 12% in the Carlevale group and 17.2% in the Artisan group. **Conclusions:** The use of the Carlevale IOL, implanted using a sclerocorneal tunnel technique, presents a valid option for treating complex lens dislocations. The scleral fixation of the Carlevale IOL minimizes risks associated with iris fixation, such as chronic inflammation and pupil distortion, making it particularly suitable for patients with damaged irises.

## 1. Introduction

When capsular support is insufficient for an intraocular lens (IOL) implantation, the two most common options are IOL fixation to the iris or to the sclera. Since the introduction of inverted retroiridial fixation of the iris-claw lens (Artisan Aphakia; OPHTEC) in 2002, multiple studies have demonstrated the effectiveness and safety of this approach [1,2,3,4,5,6].

In scleral-fixated IOLs using sutures, suture-related complications remain significant issues. Suture breaks were observed in nearly 1/3 of cases within a six-year observation period. Other complications include hypotony and IOL tilt [7].

With the introduction of the sutureless intrascleral fixation of foldable three-piece hydrophilic IOLs by Scharioth and modifications by Agawal and Yamane, new surgical alternatives became available [8,9,10]. These methods allow the sutureless fixation of the haptics in scleral tunnels parallel to the limbus. However, concerns exist regarding the long-term stability and centration of the IOL. As a development of the sutureless technique, Rossi and colleagues recently introduced the one-piece Carlevale IOL (FIL-SSF; SOLEKO). Scleral fixation is achieved via two T-shaped haptics, allowing for stable positioning in the posterior chamber, reducing the risk of decentration and “iris chafing”. Additionally, the flexible nature of the haptics allows for some adjustment of different sulcus distances. Several studies have reported favorable surgical outcomes [11,12,13,14].

This retrospective study aimed to compare the surgical and refractive outcomes using the Carlevale IOL with those of the retropupillary-fixated Artisan IOL, both implanted through a 6 mm sclerocorneal tunnel incision.

## 2. Materials and Methods

A retrospective analysis was conducted on consecutive patients diagnosed with IOL luxation/subluxation or aphakia, treated at the University Eye Clinic of Muenster between April 2023 and August 2024. The study was approved by the Ethics Committee of Westphalia-Lippe (2024-594-f-S) and adheres to the guidelines of the Declaration of Helsinki.

**Figure 1:**
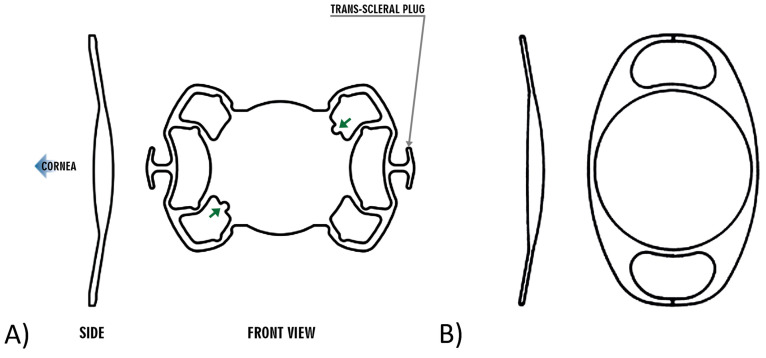
(**A**) Carlevale IOL FIL SSF (image from the Soleko product information sheet). (**B**) Artisan IOL Aphakia model 205 (image from the OPHTEC information sheet).

Two surgeons (NE, CRC), each with appropriate experience, performed the surgeries. Patients were divided into two groups based on the type of implanted IOL: Group 1 received a sutureless sclera-fixated Carlevale IOL (FIL SSF Monofocal; SOLEKO SPA, Pontecorvo, Italy), while Group 2 received a retropupillary Artisan IOL (Aphakia Model 205; OPHTEC, Groningen, The Netherlands) (Figure 1, Table 1).

Only patients for whom a 6 mm sclerocorneal tunnel was necessary for the procedure due to the following dislocated lens conditions were included: phakic Cataracta rubra, polymethylmethacrylate (PMMA) anterior chamber lenses, PMMA posterior chamber lenses, capsule-supported large lens models with or without capsular tension rings, and lenses with pronounced Soemmering’s ring. Both groups were compared in terms of age, axial length, and refractive status at baseline. Only eyes with complete preoperative ophthalmological exams and postoperative evaluations after six weeks were included in the study. Data extracted from patient records included best-corrected visual acuity (BCVA), preoperative IOL measurements, intraocular pressure (tonometry), anterior and posterior segment examinations, and fundus exams after mydriasis. The refractive predictive error (PE) was calculated as the difference between the spherical equivalent (SE) after six weeks and the expected value from biometry. Patients were classified as phakic, aphakic, or pseudophakic based on their preoperative refractive status. The surgery duration for IOL implantation was recorded from the operation reports. Postoperative complications, such as corneal edema, IOL dislocation, vitreous hemorrhage, hypotony, conjunctival erosion, and retinal detachment, were evaluated. An IOL dislocation was defined if the edge of the IOL touched the visual axis, causing a significant loss of vision. Hypotony was considered a postoperative complication when the intraocular pressure (IOP) was below 5 mmHg.

Preoperative calculation of the intraocular lenses (IOLs) was performed for both groups using the SRK/T formula (A Constant Carlevale: 119.1; Artisan: 116.9), with emmetropia being the target refraction in most cases. The surgeries were performed under general anesthesia. A 23- or 25-gauge trocar for infusion was placed temporally inferior at the start.

In the Artisan group, additional trocars were positioned nasally and temporally superior. After opening the conjunctiva, a 6 mm wide sclerocorneal tunnel was prepared about 1.5 mm behind the limbus, followed by two paracenteses at the 2-o’clock and 10-o’clock positions. The dislocated lens was removed via the sclerocorneal tunnel. To avoid vitreous prolapse or vitreous traction, vitrectomy was performed in all patients. After inserting the Artisan IOL into the anterior chamber and correct retropupillary placement, the enclavation of the haptics into the iris tissue was performed using a phaco spatula.

**Figure 2:**
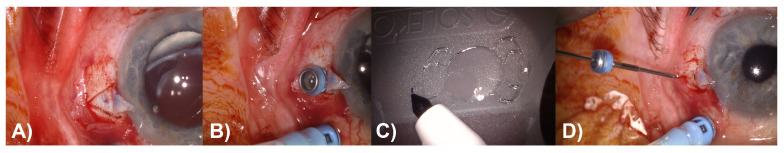
Illustration of the implantation of the Carlevale intraocular lens (IOL). (**A**) Preparation of a scleral flap and marking of the 1.8 mm distance posterior to the limbus. (**B**) Careful insertion of a 25 G trocar precisely 1.8 mm behind the limbus. (**C**) Marking of the leading IOL haptic position for accurate placement. (**D**) Externalization of the haptic using a 25 G forceps and positioning of the haptic under the prepared scleral flap for stabilization of the lens.

In the Carlevale group, corneal markings were made on the 0° and 180° axes, and the conjunctiva was opened peritomically in these areas. Two scleral flaps were prepared in line with the corneal markings. At a distance of 1.8 mm from the limbus, 2 sclerotomies were made under the scleral flaps using a 25-gauge lance, and the corresponding trocars were placed (Figure 2A,B). To ensure better visualization and reliable verification of the correct orientation of the IOL during capture and externalization of the proximal haptic, the edge of the leading transscleral plug was marked, which should be oriented superiorly after implantation (Figure 2C). The Carlevale IOL was injected into the anterior chamber using an injector through the sclerocorneal tunnel. The first haptic was grasped with forceps and externalized under the scleral flap. Then, the second haptic was grabbed using the handshake technique and also externalized (Figure 2D). Finally, the scleral flaps, sclerocorneal tunnel, and conjunctiva were closed with an absorbable 8–0 suture.

To calculate the PE, the predicted refractive outcome, corresponding to the IOL power and the SRK/T formula, was subtracted from the measured postoperative SE. A negative PE indicates a myopic result, while a positive PE indicates a hyperopic outcome compared to the expected refraction. Statistical analysis and graphical presentation of the data were performed using Prism 10.3 software (GraphPad Software, LLC, Boston, MA, USA). Unless otherwise stated, the Mann–Whitney U test was used to compare data between the two groups, and the Wilcoxon matched-pairs signed-rank test was used to compare data between pre-op and post-op findings.

## 3. Results

The average age of the patients was 72.5 ± 12.5 years, with 30 male patients (58%). The mean follow-up period was 40.9 ± 5.7 days (Table 2).

The most common indication for surgery was the subluxation or luxation of a capsular-supported IOL (79%), followed by aphakia after complicated cataract surgery (11%) (Table 3).

**Figure 3:**
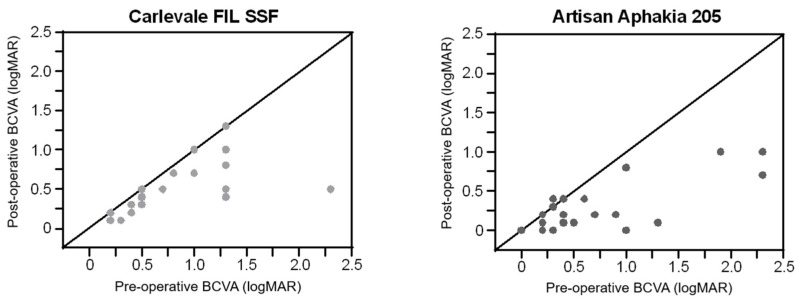
Preoperative best-corrected visual acuity (BCVA) versus postoperative BCVA (logMAR) for the Artisan group and Carlevale group. The line of equality represents no change in the BCVA, with points below the line indicating an improvement in the BCVA and points above the line indicating a decline in postoperative BCVA.

Six weeks postoperatively, visual acuity improved by 0.28 ± 0.39 logMAR in the Carlevale group (*n* = 25, *p* < 0.0001) and by 0.36 ± 0.47 logMAR in the Artisan group (*n* = 26, *p* < 0.0001). The improvement was comparable between the two groups (*p* = 0.921) (Figure 3).

There was a slight, but non-significant, increase in astigmatism in the Carlevale group from -1.17 ± 0.18 D to -1.31 ± 0.81 D (*p* = 0.265), with a change in axis from 81.5 ± 43.9° to 96.6 ± 42.8° (*p* = 0.056), and in the Artisan group from -1.31 ± 0.8 D to -1.43 ± 0.8 D (*p* = 0.855), with a change in axis from 104 ± 52° to 89 ± 48° (*p* = 0.462). There was no significant difference in the change in the postoperative cylinder between the two groups (*p* = 0.508).

**Figure 4:**
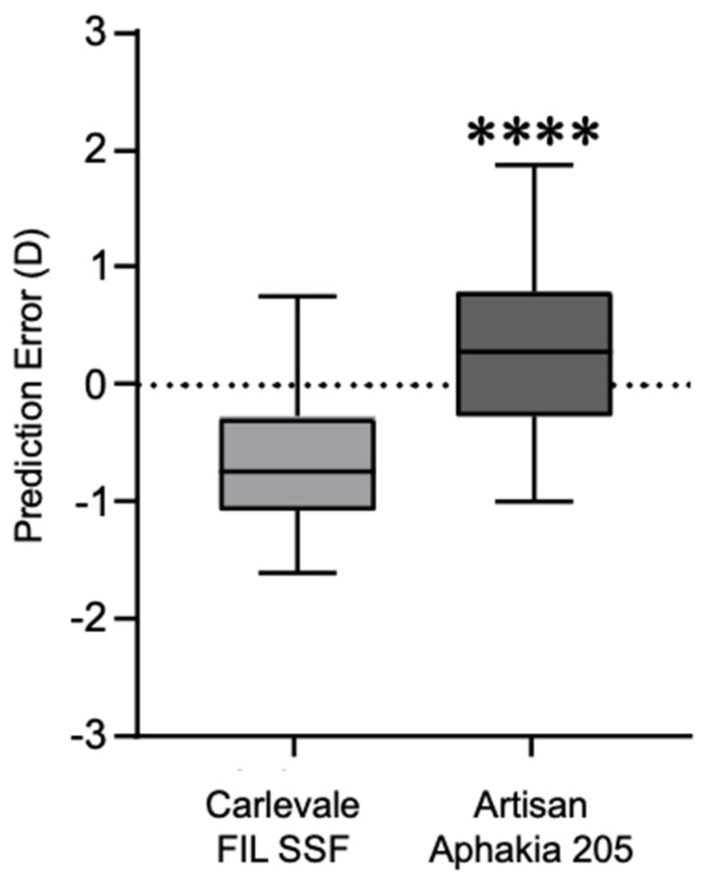
Box plots of the absolute prediction error obtained using the SRK–T formula in the Carlevale group and the Artisan group (**** = *p* < 0.0001, Mann–Whitney U test).

**Figure 5:**
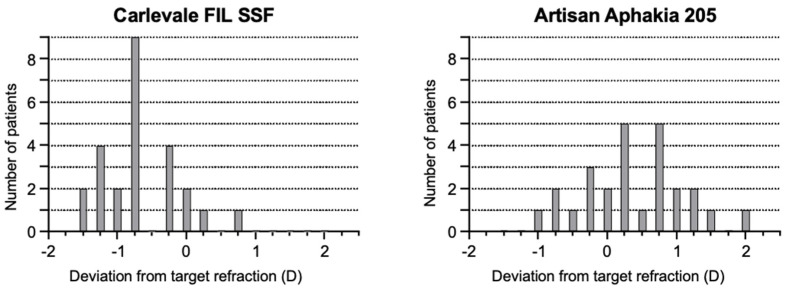
Overall distribution of the deviation from target refraction for the Carlevale group and the Artisan group.

**Figure 6:**
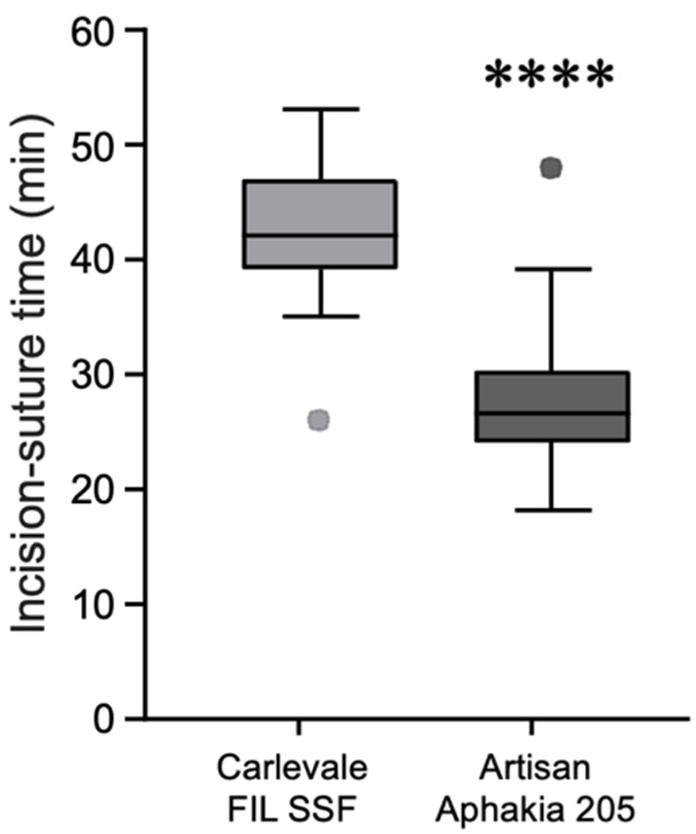
Box plots of the incision-suture times for the Carlevale FIL SSF and the Artisan Aphakia 205 IOLs (**** = *p* < 0.0001, Mann–Whitney U test).

The deviation of the postoperative prediction error was −0.67 ± 0.56 in the Carlevale group and 0.34 ± 0.71 in the Artisan group, a statistically significant difference (*p* < 0.0001) (Figure 4). Six weeks postoperatively, 28% of the Carlevale group had a deviation of a maximum of ± 0.5 D from the target refraction, compared to 42% of the cases in the Artisan group (Figure 5). The mean incision-suture time was 42.5 ± 5.8 min in the Carlevale group and 28.2 ± 6.4 min in the Artisan group (Figure 6).

In three cases (12%), the Carlevale lens flipped upside down during the implantation, necessitating the repositioning of the IOL.

Within six weeks postoperatively, an anterior chamber or vitreous hemorrhage was observed in five cases (17.2%) in the Artisan group and three cases (12%) in the Carlevale group. None of these hemorrhages was associated with IOL displacement and all resolved spontaneously without the need for additional surgery. Ocular hypotony (IOP < 5 mmHg) occurred in two cases (8%) in the Artisan group and one case (4%) in the Carlevale group, with all instances resolving on their own. In the Carlevale group, there was one case each of persistent corneal edema (4%), haptic extrusion (4%), and retinal detachment (4%).

## 4. Discussion

In this study, the surgical outcomes of the Carlevale IOL and the Artisan IOL were compared using a 6 mm sclerocorneal approach. Both techniques were found to be equally effective for treating lens dislocations, resulting in significant improvements in preoperative visual acuity. However, differences emerged in refractive outcomes and complication profiles between the two lens models. The Carlevale IOL offers stable positioning in the posterior chamber without depending on iris integrity. Its scleral fixation method reduces the risk of iris chafing and pupil distortion, issues more frequently associated with iris-claw lenses like the Artisan IOL. Although both techniques deliver similar visual acuity enhancements, the Carlevale IOL’s ability to minimize iris-related complications makes it the preferred option for patients with iris issues.

The explantation for a dislocated IOL often presents a technical challenge, requiring precise intraoperative control, especially when explanting larger lens implants or fragments. In our study, only patients requiring a sclerocorneal tunnel for IOL explantation were included. This approach offers greater flexibility during the procedure, facilitating the manipulation and intraocular use of instruments and reducing the risk of excessive tissue stretching or mechanical injury. For the Carlevale procedure, a 2.7 mm corneal incision is typically used [11]. Under challenging conditions, IOL explantation through such an incision can lead to significant manipulation of the corneal tissue. In the study by Van Severen et al., IOL explantation and the subsequent implantation of a Carlevale IOL were performed through such a corneal incision. Four weeks after the lens surgery, 13.9% of 101 cases showed persistent corneal edemas, whereas in our study, only one patient (4%) exhibited a persistent, treatment-requiring corneal edema [14].

In terms of the refractive outcomes, the Artisan group showed significantly better results, as evidenced by the lower deviation of the postoperative spherical equivalent from the preoperative target refraction (Artisan group: 0.34 ± 0.71 D compared to Carlevale group: −0.67 ± 0.56 D). These results align with the findings of other studies, where the mean deviation in the Carlevale group was −0.46 ± 1.35 D and in the Artisan group it was 0.08 ± 1.60 D (*p* = 0.019) [14].

In the Artisan group, 42% of patients achieved a target refraction between −0.5 D and +0.5 D, compared to 28% in the Carlevale group. These results also suggest a higher reliability of the Artisan IOL compared to the Carlevale IOL in terms of the final refractive outcomes. The observed variability within the Carlevale IOL cohort may be attributable to discrepancies in the interpretation of the limbus as a reference landmark. Variations in defining the limbal boundary among surgeons may lead to inconsistencies in the inter-sclerotomy distances for haptic fixation of the IOL. Such inconsistencies might also account for the pronounced postoperative myopic shift noted in the Carlevale group, aligning with the findings from previous studies. Specifically, Barca et al. [11] reported a mean prediction error of −0.24 ± 0.81 D, while Fiore et al. [12] observed a shift of −0.31 ± 0.71 D. This myopic shift was unanticipated, given that the recommended positioning for haptic externalization generally ranges between 1.5 and 2.0 mm from the limbus; in this study, a positioning of 1.8 mm was used, which would not typically be expected to induce such a refractive shift. Furthermore, subtle variations in scleral incision parameters, including symmetry and angulation, alongside anatomical variability of the ciliary sulcus, may contribute to a forward displacement of the IOL optic relative to the haptic fixation. This displacement could, in turn, modify the effective lens position, thereby influencing refractive outcomes.

Compared to other sutureless techniques, the reduced amount of tilt is a major advantage of the Carlevale IOL presumably due to the haptic design. In a comparative study by Schranz et al., the tilt was found to be lower in the Carlevale group (6.45 ± 2.03°) compared to the Yamane group (7.67 ± 3.70°) [15].

The study did not report any cases of haptic tears in the Carlevale group, a complication noted in 11% of cases in previous studies by Carlà et al. [16]. To mitigate the risk of such tears, the haptic was grasped at the outer edge of the leading T-haptic, rather than at the bar, using blunt forceps. This technique helps reduce scleral resistance during externalization. There were three eyes, in which the Carlevale IOL flipped upside down during the implantation, requiring repositioning. Maneuvering the broad Carlevale lens into the correct orientation is a challenging task. Given the convex design of the IOL, accurate intraocular alignment is essential. To ensure correct alignment during haptic capture and externalization, each haptic has a small, asymmetric incision to help the surgeon verify proper lens orientation. However, these markings are often difficult to visualize during implantation. For improved visualization and the confirmation of correct IOL positioning, marking the edge of the leading transscleral plug, which must be oriented superiorly after implantation, provides the surgeon with better clarity, particularly during lens unfolding [17].

None of the patients in the Carlevale group experienced IOL dislocations or significant decentration during the follow-up period. However, one patient developed a rhegmatogenous retinal detachment postoperatively, which was successfully managed with vitrectomy and C2F6 gas endotamponade. Importantly, the IOL remained stable during procedures such as scleral depression and vitreous substitute exchange, and no signs of C2F6 bubble migration into the anterior chamber were observed. The Carlevale IOL’s design is specifically tailored to prevent tilting, with the posteriorly angled closed haptics ensuring the optic plate remains clear of the pupillary plane, thereby reducing the risk of iris chafing. This design also acts like a spring, accommodating slight variations in the sulcus-to-sulcus distance, which further contributes to the IOL’s stability [18].

The longer incision-suture time for the Carlevale IOL implantation is due to additional steps required for this technique, including the formation of scleral flaps, precise sclerotomy creation, and the transscleral externalization of T-shaped haptics. These steps demand careful preparation to ensure secure anchoring of the IOL, which extends the procedure time.

Concepts to improve the Carlevale technique are continuously suggested such as a recent report by Giannopoulos et al. [19]. Their approach begins with the creation of two shallow scleral grooves parallel to the limbus and positioned 180° apart, serving as anchoring points for the IOL haptics. Following this, a full-thickness sclerotomy is made at the center of each groove, through which the T-shaped haptics are externalized. A major advantage of this technique is the self-sealing nature of the sclerotomies, which eliminates the need for additional sutures. By avoiding the more time-intensive preparation of scleral flaps or pockets, this method allows the scleral tissue to close naturally around the haptics, enhancing stability and simplifying the procedure.

The main limitations of our study include the small sample size and short postoperative follow-up period. A larger, multicenter study with an extended follow-up period would improve the generalizability of the results and enhance statistical power, and should be considered in future research.

In conclusion, the Carlevale IOL, implanted via the sclerocorneal tunnel technique, offers a robust and effective approach for managing complex lens dislocations. Its stable posterior chamber positioning, independent of iris integrity, makes it particularly advantageous for patients with compromised irides. The scleral fixation technique effectively reduces risks such as chafing and pupil distortion, issues more commonly seen with iris-fixated lenses, like the Artisan IOL. Although both the Carlevale and Artisan lenses provide similar visual acuity outcomes, the Carlevale IOL’s reduced risk of iris-related complications positions it as the preferred option for patients prone to anterior segment disorders or inflammation. Thus, assessing iris condition is essential in IOL selection, with the Carlevale IOL providing clear advantages for patients with compromised iris function. Despite potentially increased surgical times and some refractive variability, the Carlevale IOL remains a valuable and reliable option for complex cases.

## Figures and Tables

**Table 1:** Characteristics of Carlevale and Artisan intraocular lens.

	Carlevale IOL	Artisan IOL
Optic plate diameter (ØB)	6.5 mm	5.4 mm
Total diameter (ØT)	13.2 mm	8.5 mm
Available diopters	−5.0 D to +35.0 D	+2.0 D to +30.0 D
A Constant (optical) SRK/T	119.1	116.9

(IOL, intraocular lens).

**Table 2:** Baseline patient characteristics.

	Carlevale IOL	Artisan IOL	*p*-Value
Eyes (n)	25	26	
Age (years)	74.2 + 12.1	70.9 + 12.9	0.144 ^1^
Gender (male)	12/25	18/26	0.160 ^1^
Pre-OP Visual Acuity (logMAR)	0.79 + 0.51	0.63 + 0.65	0.070 ^1^
Pre-OP Astigmatism	−1.17 + 1.18	−1.32 + 0.78	0.233 ^1^

(^1^ Mann–Whitney U test; IOL, intraocular lens).

**Table 3:** Absolute frequencies and percentage distribution of the reasons for scleral or iris-fixated intraocular lens implantation.

Surgical Indication	*n* = 51	%
Subluxation/luxation of capsular-supported IOL	40	78
Aphakia after complicated cataract surgery	6	11
Lens luxation in phakic eye	2	4
Corneal decompensation with anterior chamber lens	2	4
Aphakia Operata	1	2

## Data Availability

Data are available upon reasonable request from the authors.
